# Hypnotics and Risk of Cancer: A Meta-Analysis of Observational Studies

**DOI:** 10.3390/medicina56100513

**Published:** 2020-10-01

**Authors:** Tzu-Rong Peng, Li-Jou Yang, Ta-Wei Wu, You-Chen Chao

**Affiliations:** 1 Department of Pharmacy, Taipei Tzu Chi Hospital, Buddhist Tzu Chi Medical Foundation, New Taipei City 23142, Taiwan; tzu.rong@tzuchi.com.tw (T.-R.P.); zero8012@gmail.com (L.-J.Y.); tawei@tzuchi.com.tw (T.-W.W.); 2 School of Pharmacy, College of Pharmacy, Taipei Medical University, Taipei City 11031, Taiwan; 3 School of Medicine, Tzu Chi University, Hualien 97004, Taiwan; 4 Department of Internal Medicine, Taipei Tzu Chi Hospital, Buddhist Tzu Chi Medical Foundation, New Taipei City 23142, Taiwan

**Keywords:** hypnotic drugs, cancer, benzodiazepines

## Abstract

*Background and objectives*: The association between hypnotic drugs and risk of cancer remains controversial. Therefore, we performed a meta-analysis to investigate this association. *Materials and Methods*: Pubmed and Embase were searched systematically to identify publications up to April 2020. The Newcastle-Ottawa scale for observational studies was used to assess the quality of studies. All included studies were evaluated by two reviewers independently; any discrepancies were resolved through discussion. *Results:* Twenty-eight studies including 22 case-control studies and 6 cohort studies with 340,614 hypnotics users and 1,828,057 non-users were included in the final analyses. Hypnotics (benzodiazepines and Z-drugs) use was significantly associated with an increased risk of cancer (odds ratio [OR] or relative risk [RR] 1.17; 95% confidence interval 1.09–1.26) in a random-effects meta-analysis of all studies. Subgroup meta-analysis by anxiolytics/sedatives effect (anxiolytics benzodiazepines vs. sedatives group (include sedatives benzodiazepines and Z-drugs)) revealed that a significant association in sedatives group (pooled OR/RR 1.26, 95% CI, 1.10–1.45), whereas no significant relationship was observed in anxiolytics benzodiazepines (pooled OR/RR 1.09, 95% CI, 0.95–1.26). Moreover, a significant dose–response relationship was observed between the use of hypnotics and the risk of cancer. *Conclusions:* This meta-analysis revealed association between use of hypnotics drugs and risk of cancer. However, the use of lower dose hypnotics and shorter duration exposed to hypnotics seemed to be not associated with an increased risk of cancer. Moreover, the use of anxiolytics effect benzodiazepines seemed to be lower risk than sedatives benzodiazepines. A high heterogeneity was observed among identified studies, and results were inconsistent in some subgroups. Randomized control trials are needed to confirm the findings in the future.

## 1. Introduction

Hypnotics (benzodiazepines and Z-drugs) are medications used to improve sleeping quality and to reduce wakefulness [1]. Benzodiazepines and Z-drugs (zolpidem and zopiclone) are common medications prescribed for sleep disorder. Moreover, benzodiazepines are prescribed in treating diseases such as seizures, anxiety, insomnia, and depression. Benzodiazepines derivatives have been distinguished into anxiolytics (i.e., diazepam, oxazepam, bromazepam, alprazepam, fludiazepam) and sedatives (i.e., flurazepam, flunitrazepam, estazolam, triazolam, temazepam, midazolam) by their effect. Z-drugs are non-benzodiazepines sedative-hypnotic medications commonly used to treat insomnia. The prevalence of benzodiazepine uses ranges from 10% to 43% worldwide among the aged population [2].

Meanwhile, most common adverse effects associated with hypnotics are residual daytime sedation, drowsiness, cognitive impairment, motor incoordination, dependence [3,4,5,6], and even tremor, which imply the possible neurotoxicity in long-term or high dose use of hypnotics. Several previous laboratory or animal studies had demonstrated that uses of benzodiazepine drugs or Z-drugs are risk factors for cancer [7].

Cancer is the second leading cause of death globally and leads to economic burden for health systems or costs for patients. Previous studies have assessed the relation between benzodiazepines or Z-drugs use and cancer risk. However, the results are still controversial. In addition, the quantitative meta-analysis has separately examined the benzodiazepines or Z-drugs and cancer risk. Until now, no published quantitative meta-analysis discussed benzodiazepines and Z-drugs long-term use conjunction with cancers. Recently, several observational studies with a larger population indicated conflicting inconsistent results. Combining the results of these studies in a meta-analysis may strengthen their statistical power. In this study, we updated the hypnotic drug correlation between use and the risk of cancer by using a meta-analysis of observational studies both case-control studies and prospective cohort studies.

## 2. Methods

### 2.1. Literature Search Strategy

PubMed and EMBASE were searched, by using selected keywords linked with benzodiazepine and non-benzodiazepine hypnotics and the risk of cancer up to April 2020. Keywords were as follows: benzodiazepine or zolpidem or zopiclone or diazepam or alprazolam or clonazepam or temazepam or oxazepam and cancer or tumor or carcinoma or neoplasm and case-control or cohort. Moreover, we reviewed the bibliographies of relevant articles to locate additional studies. The search was limited to human patients and articles in English. The detailed information on the search strategy for eligible studies is given in the flowchart provided by Preferred Reporting Items for Systematic Reviews and Meta-Analyses.

### 2.2. Selection Relevant Studies and Criteria

The studies we included meet all the following criteria: (1) case-control or cohort study, (2) investigated the associations between the use of benzodiazepines or Z-drugs and the risk of cancer, (3) outcome measures with adjusted odds ratios (OR) or relative risks (RR) and 95% confidence intervals (CI). If data were duplicated or shared in more than one study, the longest-term follow-up studies were included in the analysis. We excluded non-published studies.

### 2.3. Data Extraction

We used a standardized data extraction form that included study year, study location, study population, participant characteristics, cancer types and crude or adjusted effect sizes and their 95% confidence intervals (CIs).

### 2.4. Risk of Bias of Included Studies

Newcastle-Ottawa Scale (NOS) was used to assess the methodological quality of included case-controls and cohort studies [8]. The quality score can range from 0 to 9. A study is classified in each domain as at high or low risk according to prespecified criteria (see Table 1). All included studies were evaluated by two pharmacists (T.W.W.) and (T.R.P.) independently; any discrepancies were resolved through discussion.

### 2.5. Statistical Analyses

We conducted meta-analyses on the association of hypnotics use and the risk of cancer by observational studies. The random-effects model (DerSimonian–Laird method) was used to calculate the pooled OR or RR [9]. The Cochran *Q* test and *I*^2^ statistics were used to assess statistical heterogeneity and inconsistency. Statistical significance was set at *p* < 0.10 for Cochrane *Q* tests. Heterogeneity was considered low, moderate, or high, if the *I^2^* values was < 25%, 25–50%, and > 50%, respectively. Results were considered as statistically significant when the *p* value was less than 0.05. Publication bias was examined by using funnel plots, and Egger’s and Begg’s test was used to analyze the publication bias in our studies. A *p*-value > 0.05 based on the Egger’s and Begg’s test indicated the absence of publication bias. Statistical analysis was performed according to the Cochrane Handbook for Statistical Review of Interventions (version 5.4) [10]. The meta-analysis was performed by using RevMan software (The Cochrane Collaboration, Oxford, UK) and STATA version 15.0 (StataCorp, College Station, TX, USA).

## 3. Results

### 3.1. Study Characteristics

**Figure 1:**
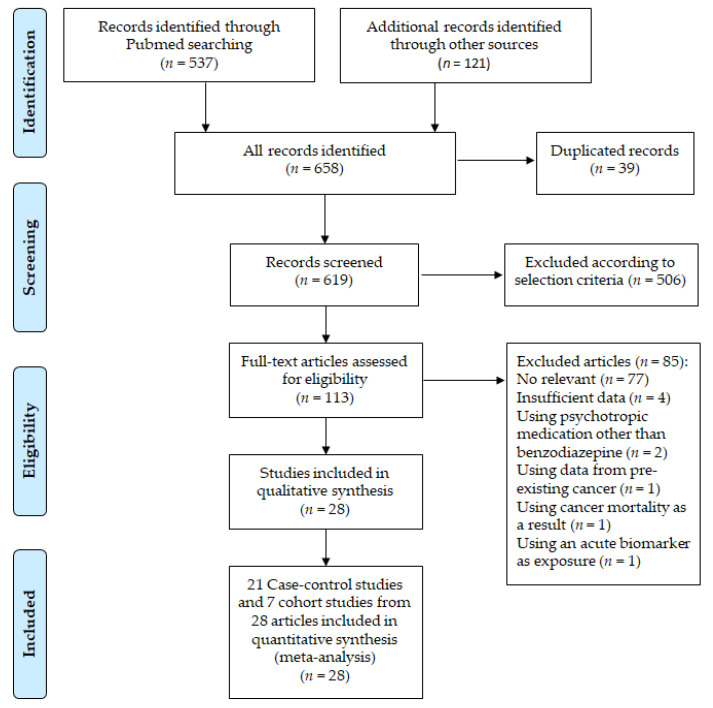
Preferred Reporting Items for Systematic Reviews and Meta-Analyses (PRISMA) 2009 flow diagram.

A total of 658 records were screened, and 113 full-text articles were assessed for eligibility. Twenty-eight articles were selected for qualitative review, including 22 case-control studies and 6 cohort studies (Figure 1). The characteristics of the 28 included studies are summarized in Table 2. The studies involved 2,168,671 participants (340,614 hypnotics users and 1,828,057 non-users).

### 3.2. Quality Assessment

We performed the methodological quality of studies based on NOS scales. The results of the methodological quality of studies are summarized in Table 2. The NOS range from 5–8; average NOS score was 6.9 and 7.1 for case-control studies and cohort studies, respectively; In case-control studies, 3 high-quality studies are included [25,29,33] (score of 8); in cohort studies, 6 high-quality studies are included [26,28,30,31,32,35] (score of 7).

### 3.3. Meta-Analysis and Subgroup Analysis

**Figure 2:**
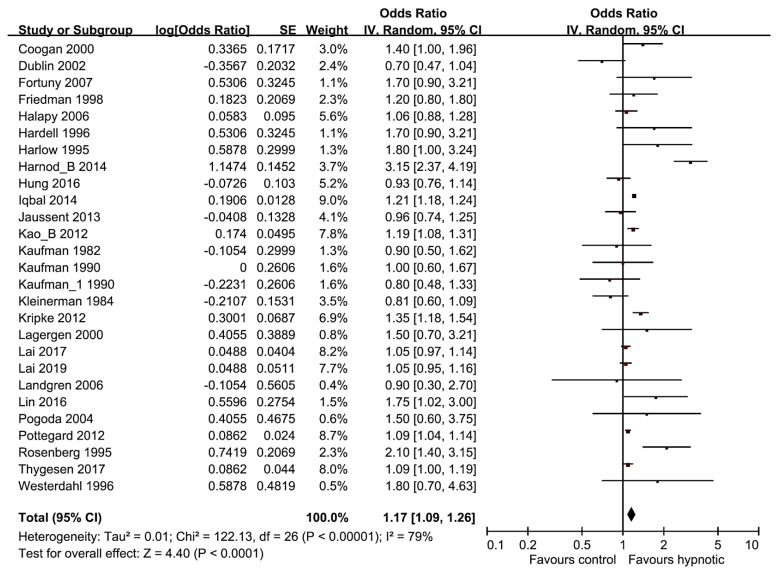
Forest plot of benzodiazepines/Z-drugs use and the risk of cancer.

Overall, the risk of cancer was greater in hypnotics user than nonusers in the random-effects meta-analysis of all 27 studies (pooled OR/RR, 1.17; 95% CI, 1.09–1.26, *p* < 0.001, *I*^2^ = 79%) (Figure 2).

**Figure 3:**
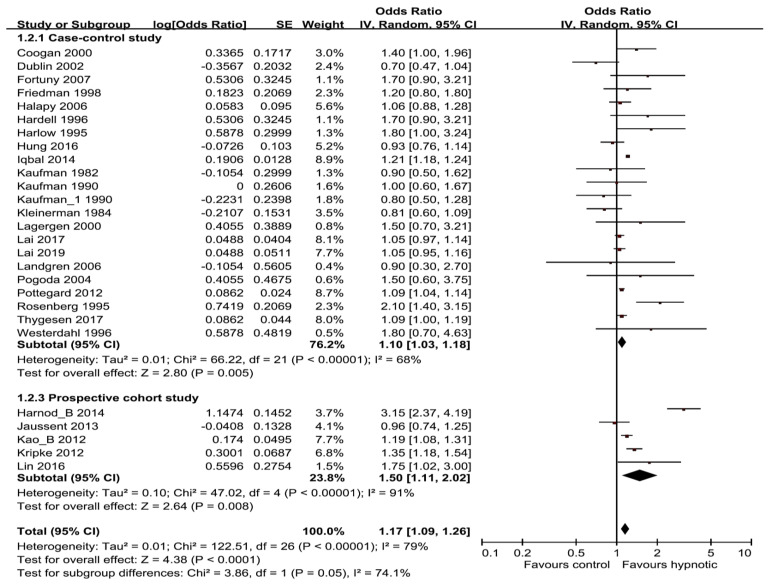
Forest plot of benzodiazepine/Z-drugs use and the risk of cancer by type of study design.

**Figure 4:**
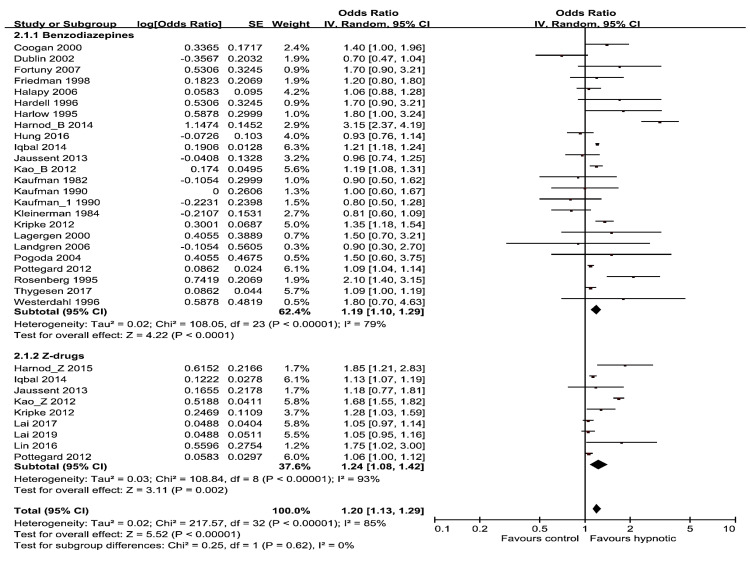
Forest plot of use of benzodiazepines vs. Z-drugs and the risk of cancer.

Subgroup analyses were carried out by research methods design (cohort or case-control study) and hypnotics categories (benzodiazepines, Z-drugs). In the subgroup analyses aligned with study design, hypnotics showed significant positive correlation with risk of cancer, both in case-control and cohort studies subgroup (pooled OR/RR was 1.10; 95% CI, 1.03–1.18 for 22 case-control studies and 1.50; 95% CI, 1.11–2.02 for 5 cohort studies) (Figure 3). In the subgroup analyses of hypnotics categories (benzodiazepines vs. Z-drugs), 24 studies are included in benzodiazepines group, the pooled OR/RR was 1.19 (95% CI, 1.10–1.29), and 8 studies are included in Z-drugs group and the pooled OR/RR was 1.24 (95% CI, 1.08–1.42) (Figure 4). Z-drugs group showed higher OR/RR than benzodiazepines group. 

### 3.4. Subgroup Meta-Analyses by Different Factors

The results of subgroup meta-analyses by different factors were showed in Table 3. The use of hypnotics was associated with an increased risk of cancer in the subgroup meta-analyses by duration of hypnotics use, cumulative yearly dose, and sedatives benzodiazepines and Z-drugs. Subgroup meta-analysis by gender showed that a significant association in both gender with 19 studies (pooled OR/RR 1.22, 95% CI, 1.13–1.32; *I*^2^ = 42%), whereas no significant relationship was observed in female with nine studies (pooled OR/RR 1.01, 95% CI, 0.89–1.14; *I*^2^ = 83%). However, there was no significant relationship observed in four studies with only elderly subjects (pooled OR/RR 1.16, 95% CI, 0.92–1.47). Subgroup meta-analysis by anxiolytics/sedatives effect (anxiolytics benzodiazepines vs. sedatives group (include sedatives benzodiazepines and Z-drugs)) revealed a significant association in sedatives group (pooled OR/RR 1.26, 95% CI, 1.10–1.45), whereas no significant relationship was observed in anxiolytics benzodiazepines (pooled OR/RR 1.09, 95% CI, 0.95–1.26). Regarding the type of hypnotics, short-acting hypnotics (midazolam, oxazepam, alprazolam, triazolam, and zolpidem) showed a significantly increased risk. Hypnotics use increased the risk of brain cancer, esophagus cancer, liver cancer, lung cancer, stomach cancer, pancreatic cancer, colon cancer, renal cancer, and prostate cancer. However, no significant association was observed in malignant melanoma, breast cancer, and ovarian cancer. When compared with nonusers of hypnotics, the pooled OR/RR for the risk of cancer was 1.03 (95% CI, 1.01–1.05) in a low dose, 1.30 (95% CI, 0.97–1.75) in a medium dose, and 2.03 (95% CI, 1.19–3.46) in a high dose (Table 3).

### 3.5. Publication Bias

**Figure 5:**
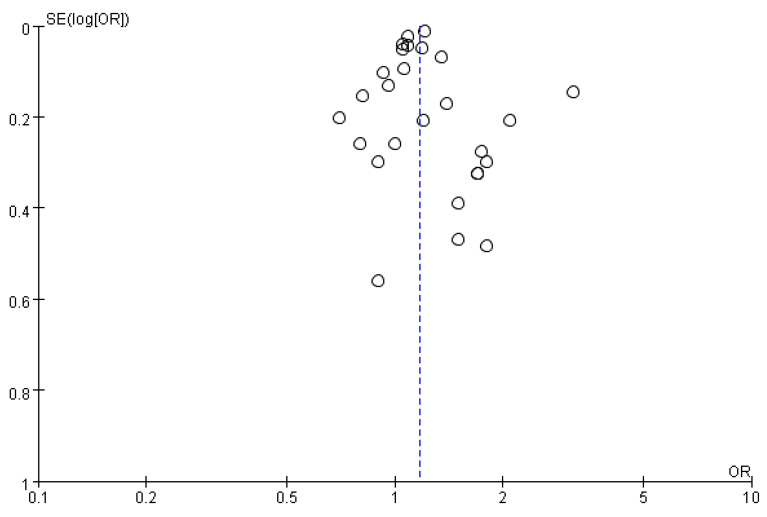
Publication bias funnel plots for use of benzodiazepines/Z-drugs and the risk of cancer.

A visual inspection of the funnel plot of OR/RR from these studies revealed asymmetry (Figure 5). However, both the Egger’s and Begg’s test suggested no statistical evidence of publication bias, with *p* value of 0.541 and 0.420, respectively.

## 4. Discussion

In our updated meta-analysis studies, the use of hypnotics was associated with an increased risk of cancer. Subgroup meta-analyses by different factors also showed similar results. However, this meta-analysis revealed that the cancer risk is related to dose–response, sedatives benzodiazepines and Z-drugs (pooled OR/RR 1.26, 95% CI, 1.10–1.45), and duration of hypnotics use (long term use: pooled OR/RR 1.11, 95% CI, 1.02–1.21). In recent years, several literatures have reported a tentative link between benzodiazepines and/or Z-drugs exposure with adverse outcomes such as respiratory disease exacerbation, infections, inflammation, dementia, pancreatitis, and cancer [39]. These kinds of adverse outcome, especially hypnotics, may relate to inflammation, infection, or cancer patients suffering from psychiatric and leading to more benzodiazepines exposure might reflect sharp of cancer diagnosed.

According to a review by Brambilla et al., the fact that the mechanisms of benzodiazepines- and Z-drugs-induced tumorigenesis remains tentative and unclear [40]. A study has reported evidence that use of hypnotics may lead to decline in immune function. Several animal studies have revealed that benzodiazepines disrupted the processes of phagocytes spreading and macrophages oxidative bursting [41,42]. These may be reduced release of the proinflammatory cytokines interleukin-6 and interleukin-13 in blood cells because of the activation of their benzodiazepine receptors [43]. Hypnotics use showed the strongest association with the risk of brain cancer in this meta-analysis (Table 3). These findings were consistent with results of Kim et al. (2017) and Zhang et al. (2017) [2,44]. The possible mechanism is that hypnotics enhance the neurotransmitter of gamma-aminobutyric acid (GABA) by interacting with the chlorine ion channel that binds to GABA receptors. The gamma-aminobutyric acid has an inhibitory neurotransmitter effect but also can regulate cell proliferation and differentiation of brain and peripheral at various stages and may participate benign tumor growth [45,46]. However, these potential mechanisms are still unproven.

Currently, there is still a lack of conclusive experimental data, but alarm signals for cancer risk have been raised by researchers for hypnotic drugs based on observational study findings [26,27,28,33]. A previous meta-analysis of 22 observational studies (18 case-control and 4 cohort studies) concluded there is an overall estimate of 19% increased cancer risk, with a significant dose–response trend, among benzodiazepines users over non-users [47]. A meta-analysis performed by Kim et al. (2017) included 6 observational epidemiological studies (3 case-control and 3 cohort studies) [44]. In a meta-analysis, compared with non-use of hypnotics, the OR for overall hypnotics (zopiclone or zolpidem) use was 1.29 for various cancers (95% confidence interval, 1.08–1.53). Our meta-analysis reported an overall estimate of 17% increased cancer risk with benzodiazepines- and Z-drugs use. Z-drugs group showed higher OR/RR (1.24, 95% CI, 1.08–1.42) than benzodiazepines group (1.19, 95% CI, 1.10–1.29). Our meta-analysis showed that hypnotics use was associated with the increased risk of cancer by duration of hypnotics, highest cumulative dose, and short acting hypnotics. According to the Bradford Hill criteria, the biological gradient (dose–response) is one of the important criteria confirming a causal relationship [48]. As shown, the dose of hypnotic is related to cancer risk, but it can also increase the causal relationship. The elderly is usually defined as individuals aged 65 years and older [49]. Insomnia is problematic for older adults. Two-fold increase was found in the intake of hypnotics among the elderly [50]. Current available hypnotic drugs all have significant risks for the elderly, such as increased risk for falls and cognitive function decline. However, we found that hypnotics use among the elderly showed a trend towards increased risk of cancer but not significantly (OR/RR, 1.16; 95% CI, 0.92–1.47; *I*^2^ = 84%). It may be associated with the small sample size (4 trials). Iqbal et al.’s (2014) study uses the Taiwanese National Health Insurance system to gather information about benzodiazepines use and cancer risk. They found that clonazepam, lorazepam, alprazolam, bromazepam, zolpidem, and zopiclone have a high risk of cancer [33]. However, we found that sedatives benzodiazepines and Z-drugs have higher cancer risk (pooled OR/RR 1.26, 95% CI, 1.10–1.45). Moreover, Z-drugs (zolpidem and zopiclone) have higher cancer risk than benzodiazepines (24% vs. 19%).

In addition, the most common risk factors for cancer including aging, smoking, alcohol consumption, family history, and exposure to chemicals or other substances [51]. The studies included in this meta-analysis, all of them, adjusted age as a confounding factor; fourteen studies adjusted alcohol drinking as a confounding factors; nine studies adjusted tobacco smoking as a confounding factor; nine studies adjusted family cancer history as a confounding factor, and only six studies adjusted medical use as a confounding factor. Thus, our studies are not excluding some important confounding factors such as alcohol drinking, tobacco smoking, family cancer history, and medical use that association between the hypnotics use and cancer risk. Nevertheless, there are still many factors that contribute to the risk factors of cancer, which have not been corrected. Therefore, the results should not be over-interpreted.

This study has some limitations. First, a portion of the included studies in our analysis did not adjust tobacco smoking, alcohol drinking, family cancer history, and medical use factors, which are known as important factors related to cancer. Second, this study only included cohort studies and case-control studies because there are no randomized controlled trials published on this topic. However, cohort studies and case-control studies have a lower level of evidence than randomized controlled trials.

## 5. Conclusions

The present meta-analysis found that hypnotics use was associated with an increased risk of cancer. However, the use of lower dose hypnotics and shorter duration exposed to hypnotics seemed to be not associated with an increased risk of cancer. Moreover, the use of anxiolytic effect benzodiazepines seemed to be lower risk than sedative benzodiazepines. Further large randomized controlled trials providing a higher level of evidence should be conducted to confirm our findings.

## Figures and Tables

**Table 1:** Methodological quality of the studies included in the final analysis (*n* = 28).

**Case-Control** **Studies (*n*** **= 21)**	**Selection**				**Comparability Control for** **Important Factor** **or Additional Factor**	**Exposure**			**Total**
	**Adequate Definition** **of Cases**	**Representativeness** **of Cases**	**Selection** **of Controls**	**Definition** **of Controls**		**Ascertainment** **of Exposure**	**Same Method** **of Ascertainment** **for Participants**	**Nonresponse** **Rate**	
Kaufman (1982)	1	1	0	0	2	1	1	0	6
Kleinerman (1984)	1	1	1	0	2	1	1	0	7
Kaufman (1990)	1	1	1	0	2	1	1	0	7
Harlow (1995)	1	1	1	0	2	1	1	0	7
Rosenberg (1995)	1	1	0	1	2	1	1	0	7
Hardell (1996)	1	1	0	0	1	1	1	0	5
Westerdahl (1996)	1	1	1	0	2	1	1	0	7
Friedman (1998)	1	1	1	0	2	1	1	0	7
Coogan (2000)	1	1	0	0	2	1	1	0	6
Lagergen (2000)	1	1	1	0	2	1	1	0	7
Dublin (2002)	1	1	1	0	2	1	1	0	7
Pogoda (2004)	1	1	1	0	1	1	1	0	6
Halapy (2006)	1	1	1	0	2	1	1	0	7
Landgren (2006)	1	1	1	0	1	1	1	0	6
Fortuny (2007)	1	1	1	0	2	1	1	1	8
Pottegard (2012)	1	1	1	1	2	1	1	0	8
Iqbal (2014)	1	1	1	1	2	1	1	0	8
Hung (2016)	1	1	1	0	2	1	1	0	7
Lai (2017)	1	1	1	0	2	1	1	0	7
Thygesen (2017)	1	1	1	0	2	1	1	0	7
Lai (2019)	1	1	1	0	2	1	1	0	7
**Cohort Studies** **(*n*** **= 7)**	**Selection**				**Comparability Control for** **Important** **Factor** **or Additional** **Factor**	**Outcome**			**Total**
	**Representativeness** **of the Exposed** **Cohort**	**Selection of the Non-Exposed Cohort**	**Ascertainment** **of Exposure**	**Outcome** **of Interest** **Was Not** **Present** **at Start of Study**		**Assessment** **of Outcome**	**Follow-up** **Long Enough** **for Outcomes** **to Occur**	**Adequacy** **of Follow-up** **of Cohorts**	
Kripke (2012)	1	1	1	1	2	1	1	0	8
Kao_B (2012)	1	1	1	1	1	1	0	0	6
Jaussent (2013)	1	1	1	1	2	1	1	0	8
Harnod_B (2014)	1	1	1	1	1	1	1	0	7
Kao_Z (2012)	1	1	1	1	1	1	1	0	7
Harnod (2015)	1	1	1	1	1	1	1	0	7
Lin (2016)	1	1	1	1	1	1	1	0	7

**Table 2:** Characteristics of included studies.

Author (year)	Study type	Country	Years Enrolled	Population (Hypnotic /Control)	Cancer Type	Definition of Hypnotic use	OR/RR (95% CI)
Kaufman (1982) [11]	Case-control	Canada, United States and Israel	1976–1980	1236/728	Breast	Diazepam ≥ 6 month vs. never-use	0.9 (0.5–1.6)
Kleinerman (1984) [12]	Case-control	United States	1973–1977	1075/1146	Breast	Diazepam ≥ 6 month vs. never-use	0.81 (0.6–1.1)
Kaufman (1990) [13]	Case-control	United States	1981–1987	3078/1931	Breast	Diazepam ≥ 6 month vs. never-use	1.0 (0.6–1.7)
Kaufman_1 (1990) [13]	Case-control	Canada	1982–1986	607/1214	Breast	Diazepam ≥ 6 month vs. never-use	0.8 (0.5–1.3)
Harlow (1995) [14]	Case-control	United States	1978–1987	450/454	Ovarian	Benzodiazepine vs. never-use	1.8 (1.0–3.1)
Rosenberg (1995) [15]	Case-control	United States	1977–1991	382/5695	Non-Hodgkin’s lymphoma	Benzodiazepine ≥ 1 month vs. never- use	2.1 (1.4–3.3)
Hardell (1996) [16]	Case-control	Sweden	1984–1986	329/658	Colon	Benzodiazepine vs. never-use	1.7 (0.9-3.3)
Westerdahl (1996) [17]	Case-control	Sweden	1988–1990	400/640	Malignant melanoma	Benzodiazepine vs. never-use	1.8 (0.7–4.4)
Friedman (1998) [18]	Case-control	United States	1991–1994	1993/2410	Colon	Diazepam ≥ 12 month vs. never-use	1.2 (0.8–1.8)
Coogan (2000) [19]	Case-control	United States	1976–1998	748/2992	Ovarian	Benzodiazepine < 12 month vs. never-use	1.4 (1.0–2.1)
Lagergen (2000) [20]	Case-control	Sweden	1995–1997	189/820	Esophageal	Benzodiazepine vs. never-use	1.5 (0.7–2.9)
Dublin (2002) [21]	Case-control	United States	1981–1997	314/790	Ovarian	Benzodiazepine < 6 month vs. never-use	0.70 (0.47–1.0)
Pogoda (2004) [22]	Case-control	United States	1987–1994	412/412	Acute myeloid leukemia	Benzodiazepine ≥ 6 month vs. never-use	1.5 (0.6–3.7)
Halapy (2006) [23]	Case-control	Canada	1996–1998	3133/3062	Breast	Benzodiazepine vs. never-use	1.06 (0.88–1.27)
Landgren (2006) [24]	Case-control	United States	1997–2002	179/691	Multiple myeloma	Benzodiazepine ≥ 6 month vs. never-use	0.9 (0.3–2.6)
Fortuny (2007) [25]	Case-control	United States	1980–2002	114/3996	Esophageal	Benzodiazepine vs. never-use	1.7 (0.9–3.1)
Kripke (2012) [26]	Prospective cohort	United States	2002–2007	2076 cases among 25,750	All cancers	Any hypnotic > 132 pill/year vs. non-users	1.35 (1.18–1.55)
Kao_B (2012) [27]	Prospective cohort	Taiwan	1996–2000	3520 cases among 119,239	All cancers	Benzodiazepine ≥ 2 month vs. non-users	1.19 (1.08–1.32)
Kao_Z (2012) [28]	Prospective cohort	Taiwan	1998–2000	1047/2924	All cancers	Zolpidem vs. never-use	1.68 (1.55–1.82)
Pottegard (2012) [29]	Case-control	Denmark	2002–2009	149360/1194729	All cancers	All benzodiazepine any related drugs (cumulative amount ≥ 500 defined daily dose) vs. never use	1.09 (1.04–1.14)
Jaussent (2013) [30]	Prospective cohort	France	1999–2011	1454 cases among 6696	All cancers	Hypnotic vs. never-use	0.96 (0.74–1.23)
Harnod_B (2014) [31]	Prospective cohort	Taiwan	2000–2009	274 cases among 62,050	Brain cancer	Benzodiazepine ≥ 2 month vs. never-use	3.15 (2.37–4.20)
Harnod_Z (2015) [32]	Prospective cohort	Taiwan	2000–2009	37810/37810	Brain cancer	Zolpidem ≥ 520 mg/year vs. never-use	1.85 (1.21–2.82)
Iqbal (2014) [33]	Case-control	Taiwan	1998–2009	42500/255000	All cancers	Benzodiazepine ≥ 2 month vs. never-use	1.21 (1.18–1.24)
Hung (2016) [34]	Case-control	Taiwan	2006–2011	1454/1448	Hepatocellular carcinoma	Clonazepam vs. never-use	0.93 (0.76–1.13)
Lin (2016) [35]	Prospective cohort	Taiwan	2002–2004	1728 cases among 6924	All cancers	Zolpidem vs. never-use	1.75 (1.02–3.0)
Lai (2017) [36]	Case-control	Taiwan	2011–2012	77986/77986	Hepatocellular carcinoma	Benzodiazepine vs. never-use	1.5 (1.45–2.44)
Thygesen (2017) [37]	Case-control	Danish	2002–2009	1854/4950	All cancers	Benzodiazepine > 500 DDD (1–5 years) vs. never-use	1.09 (1.00–1.19)
Lai (2019) [38]	Case-control	Taiwan	2000–2013	4912/4912	Colorectal	Zolpidem vs. never-use	1.05 (0.95–1.15)

Abbreviation: OR, odds ratio; RR, relative risk; CI, confidence interval; DDD, defined daily dose.

**Table 3:** Benzodiazepines/Z-drugs use and the risk of cancer in the subgroup meta-analysis by different factors.

Factors	Study Number	Summary OR or RR (95% CI)	Heterogeneity *I*^2^ (%)	Random/Fixed Effects
All	27	1.17 (1.09–1.26)	79%	Random
Region				
America	14	1.15 (0.95–1.36)	62%	Random
Europe	6	1.09 (1.05–1.14)	0%	Random
Asia	7	1.24 (1.09–1.42)	91%	Random
Type of cancer				
Brain cancer	5	1.93 (1.29–2.88)	82%	Random
Malignant melanoma	4	1.01 (0.78–1.31)	0%	Fixed
Esophagus cancer	6	1.56 (1.32–1.84)	0%	Fixed
Breast cancer	10	1.08 (0.96–1.22)	61%	Random
Liver cancer	7	1.38 (1.17–1.63)	89%	Random
Lung cancer	5	1.24 (1.04–1.48)	80%	Random
Stomach cancer	3	1.18 (1.05–1.34)	4%	Fixed
Pancreatic cancer	2	1.38 (1.20–1.58)	0%	Fixed
Colon cancer	7	1.11 (1.01–1.23)	59%	Random
Ovarian cancer	7	1.07 (0.86–1.33)	50%	Random
Renal cancer	4	1.51 (1.18–1.94)	60%	Random
Prostate cancer	4	1.29 (1.07–1.55)	70%	Random
Gender				
Female	9	1.01 (0.89–1.14)	42%	Fixed
Male and Female	19	1.22 (1.13–1.32)	83%	Random
Elderly ≥ 65	4	1.16 (0.92–1.47)	84%	Random
Anxiolytics/ Sedatives				
Anxiolytics benzodiazepines	9	1.09 (0.95–1.26)	30%	Random
Sedatives benzodiazepines and Z-drugs	10	1.26 (1.10–1.45)	93%	Random
Duration of hypnotics use				
<6 months	12	1.03 (1.02–1.04)	35%	Fixed
≥6 months	13	1.05 (1.02–1.08)	0%	Fixed
≥5 years	9	1.11 (1.02–1.21)	0%	Fixed
Cumulative yearly dose				
Lower	5	1.03 (1.01–1.05)	10%	Fixed
Moderate	6	1.30 (0.97–1.75)	95%	Random
Highest	6	2.03 (1.19–3.46)	97%	Random
Type of hypnotics				
Long-acting (Diazepam)	8	0.97 (0.93–1.01)	37%	Fixed
Intermediate-acting	4	1.21 (0.93–1.57)	88%	Random
Short-acting	9	1.29 (1.12–1.48)	92%	Random
Methodological quality				
High quality	19	1.14 (1.04–1.25)	92%	Random
Low quality	10	1.59 (1.27–1.98)	85%	Random
